# General Practice Staff and Consumer Views of Voluntary Patient Registration in Australia Under the MyMedicare Scheme: A Qualitative Study

**DOI:** 10.5694/mja2.70318

**Published:** 2026-10-30

**Authors:** Sanjyot Vagholkar, Ashfaq Chauhan, Gaston Arnolda, Maha Iqbal, Janani Mahadeva, Rebecca Mitchell, Elizabeth Manias, Kate Churruca, Bronwyn Newman, Magdalena Raban, Kirsten Moore, Ramya Walsan, Johanna Westbrook, Jeffrey Braithwaite, Charbel Badr, Lily Edwards, Reema Harrison

**Affiliations:** ^1^ Medical Program Macquarie University Sydney New South Wales Australia; ^2^ General Practice Clinic Macquarie University Health Sydney New South Wales Australia; ^3^ Australian Institute of Health Innovation Macquarie University Sydney New South Wales Australia; ^4^ Monash Nursing and Midwifery Monash University Melbourne Victoria Australia; ^5^ Australian Primary Health Care Nurses Association Melbourne Victoria Australia; ^6^ Hunters Hill Medical Practice Sydney New South Wales Australia; ^7^ Royal Australian College of General Practitioners Melbourne Victoria Australia; ^8^ Ingham Institute for Applied Medical Research Sydney New South Wales Australia

**Keywords:** continuity of patient care, general practice, health services research, health systems, physician–patient relations, primary care

## Abstract

**Objectives:**

To understand general practice staff and consumers' views of MyMedicare, which is Australia's voluntary patient registration scheme introduced in October 2023 to enhance continuity of care.

**Study Type:**

Qualitative exploratory research using semi‐structured interviews. Analysis was conducted using an inductive approach.

**Setting, Participants:**

Australian primary care including general practices, community organisations and the wider Australian community. Data were collected from January 2025 to April 2026. Consumers eligible to register with the MyMedicare scheme and general practice staff (general practitioners, practice nurses, practice managers, reception staff) involved with registering patients under the scheme. Interviews conducted with 45 general practice staff and 50 consumers.

**Main Outcome Measures:**

Qualitative expression of general practice staff and consumers' awareness of, and knowledge about, MyMedicare, their perceptions of the value of the scheme and practice staff's experiences of implementing the MyMedicare scheme.

**Results:**

Staff and consumers demonstrated broad support for the concept of voluntary patient registration to enhance continuity of care; however, staff described limited current benefits, substantial implementation burden for practices and administrative processes, which could sometimes distract from clinical work and undermine continuity of care. Consumers had limited understanding of the MyMedicare scheme and why they should register and, in most instances, did not find it was providing them with anything beyond their pre‐existing care.

**Conclusion:**

The current paucity of benefits from MyMedicare registration for both consumers and practices may have implications for the success of the scheme. Anticipated funding for patient care linked to MyMedicare has encouraged large numbers of practices to register despite the administrative workload, whereas low consumer awareness of the scheme and limited benefits for patients are likely contributing to much lower levels of patient registration. Without further incentives for consumers and practices, the MyMedicare scheme may have limited impact on continuity of care and patient outcomes.

## Plain Language Summary

**The known:** Voluntary patient registration in Australian general practice was introduced in October 2023 under the MyMedicare scheme. It aimed to encourage continuity of care for patients with a nominated practice.

**The new:** General practice staff and consumers support the concept of the scheme, but have found that MyMedicare is poorly communicated, presents a substantial administrative burden to practice workload and provides limited benefits for patients and practices in its current form.

**The implications:** There may be limited impact on continuity of care without evolution of the MyMedicare scheme to provide further tangible benefits for patients and practices.

## 1 Introduction

In Australia, 76.9% of the adult population nominated that they had a preferred general practitioner (GP) in 2022–2023 [1], but there was no formal recognition of this relationship. The MyMedicare scheme, which is the voluntary patient registration scheme in Australian general practice, was introduced in October 2023, following recommendations of the Strengthening Medicare Taskforce Report [2]. The scheme aims to formalise the relationship between a patient, their GP and practice team to encourage continuity of care, which is associated with better patient outcomes [3]. In order to participate in the scheme, practices and GPs must register for MyMedicare via Services Australia. Following this, their patients can register with their nominated GP at the practice [4]. Registration with a practice provides benefits to patients, such as access to a Medicare (Australia's universal health insurance scheme) rebate for long (≥ 20 min) telephone consultations. Patient registration also links provision of chronic disease management plans, which provide patients with access to allied health items and nursing support, to that practice [4]. For patients living in residential aged care homes, registration enables providers and practices to access incentive payments (the General Practice in Aged Care Incentive [GPACI]) if patients receive regular care from their GP [5]. MyMedicare also provides a platform for future funding of patient programs.

Internationally, primary care systems in Europe, Canada and New Zealand have used patient registration models in primary care, with marked variation between schemes, particularly in relation to whether they were voluntary and what incentives were provided [6, 7]. The most well‐known is the National Health Service in the United Kingdom, where patients voluntarily register with a practice and must undertake all their primary care with that practice. Practices then receive funding based on their practice population. In Australia, the MyMedicare scheme is also voluntary, but registered patients are free to seek care at other practices. GPs and professional bodies, such as the Royal Australian College of General Practitioners and the Australian Medical Association, are in favour of programs that enhance continuity of care but are less supportive of capitation being part of the funding mix to support this [8, 9]. Previous trials of patient registration in Australian general practice, such as the Health Care Homes program, showed limited success [10]. The Health Care Homes program demonstrated greater access to and improved processes of care for people with chronic disease, but did not show changes in diabetes outcomes, hospital use or risk of death [11].

Understanding how patients and practices have engaged with the MyMedicare scheme would assist in identifying facilitators and barriers to implementation and uptake of voluntary patient registration in Australia [6]. This qualitative study, which is part of a wider evaluation of the MyMedicare scheme [12], aimed to understand general practice staff and consumers' awareness of and knowledge about MyMedicare, comprehend their views about benefits of participating in the scheme and explore the experiences of practice staff implementing patient registration.

## 2 Methods

### 2.1 Study Design

This study was conducted as qualitative exploratory research using semi‐structured interviews. An inductive approach using the principles of thematic analysis was chosen to understand the experiences of participants with the MyMedicare scheme [13]. The study was reported according to the COREQ standards for qualitative research (Table S1).

### 2.2 Setting

Australian primary care stakeholders directly interacting with, or eligible to interact with the MyMedicare scheme, including consumers, GPs, general practice nurses and administrative staff were eligible to participate in the study. Data were collected from January 2025 to April 2026.

### 2.3 Participant Recruitment

Potential participants were consumers (patients, carers, general community) and GPs, general practice nurses and staff with responsibility or oversight of registering patients with the MyMedicare scheme in Australian general practice [14]. Consumers did not need prior knowledge of MyMedicare and did not have to be registered with MyMedicare to participate.

Study recruitment occurred through advertisements sent to relevant professional bodies, consumer organisations, primary health networks, general practice‐based research networks and institutional social media accounts of the research team. Respondents were purposively sampled to ensure representation from different geographical locations. Potential participants completed an online form to indicate their interest and were contacted by the research team to complete the informed consent process and arrange an interview.

### 2.4 Data Collection

Interviews were conducted by members of the research team (MI, JM, AC, GA) who were experienced in interview data collection. Each interview was held online via Microsoft Teams or Zoom platforms, or via telephone, and lasted 30–60 min. Interviewers guided participants through a semi‐structured interview schedule informed by the Theoretical Framework of Acceptability [15] and the Consolidated Framework for Implementation Research [16]. Interview schedules for healthcare staff and consumers (Sections S1 and S2) were piloted with two practising GPs and two consumers, respectively, and refined ahead of use. Participation was voluntary, with consumers and practices offered remuneration via a gift card. Interviews were audio‐recorded and transcribed using the Microsoft Word, Teams or Zoom transcribing functions.

### 2.5 Data Analysis

Inductive coding of interview transcripts was undertaken by two researchers (AC, RH) using NVIVO software. This involved detailed reading of the raw data to develop preliminary codes. Through discussion with the wider research team, the derived codes were developed into themes [13]. The researchers returned to the raw data where there was uncertainty in order to ensure that the findings reflected the data. Differences in interpretation were resolved by team discussion.

### 2.6 Research Team and Reflexivity

The research team was a mix of clinician and health systems researchers: three female GPs, a male allied health professional and two female and one male health systems researchers. Six members of the team hold PhD qualifications. The GP researchers had personal experience of MyMedicare in practice, which provided a deep understanding of the implementation process. This raised the possibility of bias in interpreting findings; however, the involvement of non‐clinical researchers facilitated a balanced approach.

### 2.7 Ethics Statement

Ethics approval for the study was obtained from the Macquarie University Human Research Ethics Committee (Project ID: 520241843160560).

## 3 Results

A total of 95 participants (50 consumers and 45 general practice staff) completed the interviews. Demographic information for participants is shown in Tables 1 and 2. The 50 consumers were aged 36–85 years and 37 had at least one chronic condition. The 45 general practice staff included GPs, practice nurses, practice managers and reception staff from 22 GP‐owned general practices.

**TABLE 1:** Characteristics of consumer participants.

Characteristics	*N* (%)
Total number of consumers interviewed	50
Age range, years	
20–39	3 (6%)
40–59	18 (36%)
60–79	22 (44%)
≥ 80	1 (2%)
Not recorded	6 (12%)
Sex	
Female	35 (70%)
Male	14 (28%)
Non‐binary	1 (2%)
State	
New South Wales	26 (52%)
Victoria	12 (24%)
South Australia	7 (14%)
Queensland	2 (4%)
Australian Capital Territory	2 (4%)
Tasmania	1 (2%)
Geographical classification	
Metro	34 (68%)
Regional/remote	15 (30%)
Not recorded	1 (2%)
Enrolled into MyMedicare	
Yes	14 (28%)
No	23 (46%)
Unsure	13 (26%)
Categories^a^	
Any chronic condition	37 (74%)
Cancer	12 (24%)
Arthritis/chronic pain	11 (22%)
Diabetes	7 (14%)
Anxiety/depression/PTSD	7 (14%)
People with disability	4 (8%)
Carers	3 (6%)

Abbreviation: PTSD, post‐traumatic stress disorder.

^a^ Some individuals are in multiple categories.

**TABLE 2:** Characteristics of healthcare provider participants and practices.

Characteristic	*N* (%)
*Total number of healthcare providers*	45
Provider role	
General practitioner	14 (31%)
Practice nurse	9 (20%)
Practice manager	14 (31%)
Reception staff	8 (18%)
*Total number of practices*	22
State	
New South Wales	9 (41%)
Victoria	4 (18%)
Queensland	5 (23%)
Tasmania	3 (14%)
South Australia	1 (5%)
MMM^a^ group	
1–2	17 (77%)
3–4	3 (14%)
5–7	2 (9%)

Abbreviation: MMM, Modified Monash Model.

^a^ Modified Monash Model geographical location. Modified Monash (MM) Category 1–7: MM1 is a major city and MM7 is a very remote area.

Our analysis identified four themes regarding the MyMedicare initiative: (i) conceptual support for promoting continuity of care; (ii) knowledge gaps about the scheme and long‐term implications; (iii) limited current tangible benefits and expectations of future gains; and (iv) considerable burden to practices. Further supporting quotes are provided in Table 3.

**TABLE 3:** Themes and supporting quotations.

Theme	Sub‐theme	Supporting quotes
Conceptual support for continuity of care		‘… my understanding is that if you had multiple conditions, that that was the idea of MyMedicare.’ [CON5] ‘… we do know each other (GP and patient), so I have that, in a way, that head start, where I'm not having to explain myself to a new person (GP) the whole time. And that's where developing a relationship with a GP would be a really beneficial, I think. How MyMedicare encourages people to do that, I can't really say it. I haven't seen much information about it.’ [CON45] ‘… there is communication with the primary, the patient's chosen primary care provider.’ [GP12] ‘… all that we've seen is the GP aged care incentive being implemented. But unfortunately, I think for most GPs, that's actually been a deterrent to providing aged care services rather than encouraging aged care services.’ [GP2]
Knowledge gaps about the scheme and long‐term implications		‘… I don't understand [what were] the reasonings on how it would benefit our local patients other than now with the longer telehealth where the doctor gets more benefit.’ [PM13] ‘… I was thinking it would be more allowing others outside that primary carer so they could all add their little bits in, which would probably be a nightmare to try and do because everyone's got different computer systems or different software. Yes, … it would be a benefit, but I assumed it was something that happened anyway.’ [CON18] ‘I think there's massive potential for good things to come from it in terms of supporting patients to be able to access what they need and have that centralised in some sort of system to make sure that particularly, you know, GPs are actually getting the information they need to be able to actually provide best care for patients.’ [GP12]
Limited current tangible benefits and expectations of future gains		‘I thought it was going to give me a link to potentially help my medical team access my medical records. I thought it was more to do with that when I started.’ [CON 45] ‘We wanted to be able to use the item numbers for longer telehealth. So yes, we have been encouraging them (patient registration).’ [PM3] ‘Those (telehealth) consults would go 30 to 40 min because you're trying to explain what the condition is with an interpreter and then an interpreter will come back with another question … So yeah, it would go for 30 min and we would not be paid for that service. But the good thing now is that we are paid for what we were doing.’ [GP9] ‘Yeah, we probably had two dozen people that won't sign it because there's no really benefit to them. And they said that I have long telehealth anyway, so that I don't need to.’ [PM13] ‘… when it comes to us because we use interpreters for most of our consults. So, and part of it, we do as doctors, GPs keep that MyMedicare paperwork here, try and explain that to them (refugee populations) and the usefulness of it.’ [GP9] ‘There [are] anticipated changes to the Medicare item numbers for chronic disease management, but nobody seems to be able to give us any information about exactly what that looks like in terms of dollar figures.’ [GP2] ‘I think at the moment there's way too much bureaucracy, for example, with the aged care incentive and not nearly enough funding associated with it given the value it delivers to government.’ [GP15] ‘I don't see the purpose, you can't track. If it's a matter of tracking the patient's care or ensuring the patient gets adequate care at any practice, I don't think it's the right mechanism.’ [PM8]
Considerable burden to practices	Patient education and support	‘But I'd have to say I have really never seen any advertising.’ [CON15] ‘… the nurse and the receptionist are helping with the discussion of MyMedicare at the reception and ask patients to fill in the forms. Yeah, so they're doing the bulk of it to be honest of registering patients. And we tried, we tried doctors doing a bit. We kept forgetting.’ [GP8]
	Disruption to continuity for patients who transfer registration	‘We found ourselves in a situation where a patient is a regular loyal patient but they may have … seen … someone else at some stage, maybe it was when they were on holidays or if they had to attend another surgery when we were closed and some completely random doctor will take an opportunity to do a care plan for them and even though our doctor's the one providing continuous care for that patient, they do not get the benefit of those higher rebates.’ [PM3] ‘But we get [care plans] rejected all the time from children, and the parents don't even know they've been registered somewhere else.’ [NS7] [How often would you get a MyMedicare item rejected?] ‘I think if our admin team wasn't proactive in double‐checking, it would be daily.’ [PM14]
	Tensions for multi‐site or relocating practices	‘… we can only bill the patient at their very specific clinic they've been seen at …even if their doctor works at both. So, our clinic is a bit different to others. Um, where a patient might have that half an hour phone call at the wrong clinic and we can't bill them.’ [REC2] ‘… [Registrars] might do 6 months here … and then go to [Site 2] for another 6 months … but I still have to transfer their … registration and I have to get consent to do that … they've got to fill in another form and I've got to, you know, explain and get it all done.’ [PM11]
	Manual processes and opportunity costs	‘If we identify them as not having MyMedicare already, we prompt them to do a registration online … Even if they come through by HotDoc, we still have to log them through PRODA … It's a really terrible logistical nightmare, unfortunately.’ [PM3] ‘We did have a couple of complaints from patients that even though they were registered, they were still getting prompted by HotDoc and that a lot of them had trouble logging in through their MyGov account.’ [PM3] ‘… they come to us, you know, to check in, we check their files, see if they're registered. If not, we offer them the form. Explain the form to them. They sit down, sign it, give it back to us.’ [PM10] ‘I think at the moment the main barrier is time. It does take a few minutes extra to talk through the system with every patient that you see and actually enrol them.’ [GP12] ‘Even with the aged care thing, we get told you don't have to do anything because patients are already enrolled. And then after it starts, ‘oh sorry guys, we didn't really mean that, you need to enrol them again’. So you have to enrol your enrolled patient in MyMedicare to get the aged care incentive to you. So instead of wasting your time once, you're wasting your time twice.’ [GP6]

Abbreviations: CON, consumer, GP, general practitioner; NS, practice nurse; PM, practice manager; PRODA, Provider Digital Access; REC, receptionist.

### 3.1 Conceptual Support for Promoting Continuity of Care

Both practice staff and consumers expressed conceptual support for MyMedicare as a mechanism to promote continuity of care. Practice staff articulated alignment between the goals of MyMedicare and long‐standing professional principles, particularly relational and management continuity between patients and clinical teams.

… so there's a relationship between the practice and the patient, hoping to lead to sort of better patient care, better organisation of care, better facilitation. [GP1]

Consumers also acknowledged that having a recognised relationship with a GP was good for their care.

… so that is obviously a good thing to do, to sort of maintain a relationship with one GP, so that's good. [Consumer (CON)50]

Practice staff commented that registration could strengthen accountability and clarity around which practice held primary responsibility for a patient's care. However, support for the concept did not equate to endorsement of its current form. Staff expressed frustration with the implementation of MyMedicare and did not find that the scheme had translated into meaningful improvements in clinical care delivery.

### 3.2 Knowledge Gaps About the Scheme and Long‐Term Implications

There was variable knowledge of the MyMedicare scheme among all participants, with few consumers being aware of the scheme. Consumers expressed confusion given the name being similar to Medicare and My Health Record (the national electronic patient record).

I don't understand the difference between the Medicare that I've been using for 40 years and this one called MyMedicare. [CON50]

For staff, knowledge gaps were evident about MyMedicare's specific features and how it was intended to support continuity of care.

To be honest with you, it's still something I don't fully understand. I know that it, like if someone's registered for MyMedicare, it can open up certain items like for longer consults (telehealth); other than that, I don't really understand a huge amount about it. [GP5]

Some clinicians and practice managers reported a clear understanding of the policy rationale but could not see how MyMedicare would enhance continuity of care. They reported that they already had well‐established relationships with most of their patients, particularly in rural areas. There was also some concern about what implications the scheme had for the long‐term funding model for general practice.

… my concern was that I didn't want to see it as a sort of capitation model here in Australia, where people lost that capacity to choose where they access care and who they choose to see for services. [GP12]

### 3.3 Limited Current Tangible Benefits and Expectations of Future Gains

A key barrier to engagement for all stakeholders was the perception that MyMedicare currently offers few benefits for practices, staff or patients.

It was really hard to generate enthusiasm among any of our GPs, any of our practice nurses and any of our reception staff because nobody could see any tangible benefits coming out of it. [GP2]

Although acknowledging that additional incentives or programs may be linked to MyMedicare in the future, many staff characterised the current phase as one of significant administrative work with minimal immediate return. Some consumers felt there was no change in service delivery as a result of registration, as they already had an established relationship with their GP.

But I am not sure what benefit I am getting [the] last 2 years and this year there is no difference with any follow‐up with doctor. Not getting any telephone consults. [CON10]

Practices felt compelled to participate to avoid exclusion from future funding initiatives. This perception contributed to cautious engagement rather than enthusiasm for the existing scheme. An exception noted by some participants was the current telehealth incentive (rebate for telephone consult ≥ 20 min), which was improving access to care for MyMedicare‐registered patients who require interpreters or who have complex needs. Staff described hope that registration might support more coordinated funding or service delivery for these groups. However, they emphasised that these initiatives had not yet materialised.

### 3.4 Considerable Burden to Practices

Practice staff consistently described the administrative burden of MyMedicare as substantial, particularly because practices perceived that they bore the primary responsibility for registering patients. They reported few patients self‐registered, resulting in practices taking on extensive education and administrative tasks. Nurses and practice managers were most frequently identified as absorbing this workload. Registering patients from residential aged care homes was time‐consuming due to the added complexity of patients with cognitive impairment and the need to obtain consent from family or guardians. The four key areas that created burden are: (i) patient education and support; (ii) disruption to continuity for dual‐registered patients; (iii) tensions for multi‐site practices; and (iv) manual processes and opportunity costs.

#### 3.4.1 Patient Education and Support

Practices reported extensive effort required to explain MyMedicare to patients, assist with registration and address confusion or mistrust. Few tangible benefits created difficulty in conveying the scheme's purpose. This burden was especially pronounced for older people and for patients with limited digital literacy, language barriers or cognitive impairment. Staff noted that supporting these groups was time‐consuming and often required focus on administrative registration requirements, thus distracting from clinical care.

#### 3.4.2 Disruption to Continuity for Dual‐Registered Patients

Staff reported challenges managing patients who had completed registration at more than one practice. Rejected Medicare billing for chronic disease management plans for regular patients somehow registered with another practice were described as disruptive to continuity of care and had a negative financial impact. Clinicians expressed frustration that administrative issues with MyMedicare registration could potentially override established relationships and fragment patient care.

… patients haven't realised that they've signed up to other practices and so they can't come to us for that continuation of care for their general health assessments and everything. [Practice manager 9]

#### 3.4.3 Tensions for Multi‐Site Practices

Practices that operated across more than one physical site identified structural incompatibilities between MyMedicare and their care models. The requirement for registration at a single physical address was described as incongruent with accessing care across multiple sites within the same organisation. Staff also highlighted additional complexity when practices relocated, requiring updates to registration details.

One of the biggest issues we faced was when we moved from one location to another, all those patients that were registered at our previous location had to be re‐registered. [Receptionist 7]

#### 3.4.4 Manual Processes and Opportunity Costs

Determining whether patients were already registered with a practice was described as a largely manual process, adding to administrative workload. Staff usually accessed this information through MyHealth Record, which is restricted to viewing by GPs or nurses. This limitation resulted in opportunity costs, with clinical staff required to undertake administrative checks that could otherwise be delegated.

## 4 Discussion

General practice staff and consumers interviewed for this study supported the concept of voluntary patient registration to enhance continuity of care. The rollout and implementation process of the MyMedicare scheme, however, was found to be confusing and burdensome. The scheme was not well communicated to consumers and the process of registration created a substantial workload for practices. Most importantly, both practice staff and consumers found there were limited benefits, financial or otherwise, for patients and practices in its current form.

Staff and consumers' positive views about MyMedicare's goal of encouraging continuity of care aligned with previous studies. Internationally, consumer surveys have demonstrated over 90% support for continuity of care in North America [17] and The Netherlands [18]. Similarly, a large multi‐country survey found that primary care physicians overwhelmingly considered relational and informational continuity to be important to quality patient care [19]. Reaction to the Australian Health Care Homes pilot revealed similar local support for activities that may improve continuity of care [20, 21].

General practice staff reported that they already had longitudinal relationships with most of their patients, reflecting previous findings that 76.9% of adults had a preferred GP and over 90% have a usual practice [1, 22]. Patient registration schemes overseas have demonstrated that it is often patients who already have a regular GP that enrol in these schemes and they do little to increase continuity of care [6]. The only clear‐cut patient incentive for MyMedicare enrolment related to long telephone consults, with the benefit of this being greater for sub‐groups, such as those with long distances to travel or those requiring interpreter services. Given the few tangible benefits for patients who register, it is not surprising that after 18 months of operation; fewer than 10% of Australians had enrolled into MyMedicare [23]. Internationally, schemes with weaker incentives have been shown to have lower enrolment rates [6].

General practice staff have over time developed an understanding of the intended purpose of MyMedicare but are also aware that the only clear‐cut benefit for practices is in telehealth, with care planning available regardless of registration [24] and GPACI replacing an existing aged care incentives program. Practices in our study reported signing up because they anticipated future funding would be linked to MyMedicare participation [25]. Over 70% of eligible practices registered in the first 7 months of scheme operation [26], suggesting that this was a commonly held belief, and aligns with overseas experience that practices are likely to register if encouraged by the payment model [6].

The considerable administrative burden of registering patients, with no specific support provided to practices for this work, is clearly a major frustration for providers. The problems highlighted by practices, such as challenges of multi‐site practices and patients unknowingly being registered at another practice, can result in rejected Medicare billings. This has financial implications for practices and patients, who may miss out on subsidised allied health visits. Therefore, a scheme that is meant to enhance patient care can inadvertently have a negative impact on service provision.

The Strengthening Taskforce Report stated that any patient registration system introduced needed to be simple, streamlined and efficient [2]. Our findings suggest this is currently not the case. MyMedicare would benefit from a reduction in the administrative load borne by practices. Mass media education and simpler processes for patients to register could reduce the load for practices; further, a clear message that MyMedicare seeks to encourage longitudinal care with a patient's usual provider may reduce the number of patients who inadvertently change practice registration. Policy design also needs to account for the possibility that some patient registration transfers reflect opportunistic attempts to capture the Medicare income associated with care plan preparation for patients. The resulting disruption to longitudinal relationships works against improving continuity of care.

Commentary before the introduction of MyMedicare concluded that program success would depend on the incentives available to practices and patients [27]. Novel approaches may be needed to incentivise patient registration and to compensate practices for the additional administrative burdens. In care planning, for example, doctors could be afforded greater discretion in managing MyMedicare patients. GPs could be allowed to allocate a broader range or a larger number of Medicare‐subsidised allied health referrals for their MyMedicare patients to incentivise patients to invest in a longitudinal relationship.

### 4.1 Limitations

An important limitation of our results is that practices that volunteered to participate in our study were all GP‐owned. We cannot therefore comment on the experiences of corporate practices, which are estimated to represent roughly one‐fifth of all Australian general practices [28, 29]. In a similar vein, there were no consumers from residential aged care homes, so we are unable to comment on their experiences with patient registration. The study did have a large number of participants representing a diverse range of consumers and practices, which was a strength and allowed for data saturation, but it is acknowledged that, given the voluntary nature of participation, there may be perspectives that are not reflected in our findings.

## 5 Conclusion

General practice staff and consumers supported the concept of a program that aimed to enhance continuity of care. However, the rollout and implementation process of voluntary patient registration via MyMedicare was found to be poorly communicated and administratively onerous and it was unclear how it would improve levels of continuity of care from what currently exists. Staff and consumers perceived few benefits for patients and practices in its current form. Anticipated funding for patient care linked to MyMedicare has encouraged large numbers of practices to register, but low consumer awareness and limited benefits for patients are likely contributing to much lower levels of patient registration. Without further incentives for consumers and practices, the MyMedicare scheme may have a limited impact on continuity of care and patient outcomes.

## Author Contributions

**Sanjyot Vagholkar:** conceptualisation, analysis, methodology, funding acquisition, project administration, supervision, writing original draft and review – editing. **Ashfaq Chauhan:** data curation, resources, investigation, analysis, methodology, project administration, visualisation, writing original draft and review – editing. **Gaston Arnolda:** data curation, resources, investigation, analysis, methodology, visualisation, writing original draft and review – editing. **Maha Iqbal:** methodology, investigation, analysis, writing review – edit article. **Janani Mahadeva:** methodology, investigation, analysis, writing review – edit article. **Rebecca Mitchell:** conceptualisation, analysis, methodology, writing review – edit article. **Elizabeth Manias:** writing review – edit article. **Kate Churruca:** writing review – edit article. **Bronwyn Newman:** investigation, writing review – edit article. **Magdalena Raban:** writing review – edit article. **Kirsten Moore:** writing review – edit article. **Ramya Walsan:** writing review – edit article. **Johanna Westbrook:** writing review – edit article. **Jeffrey Braithwaite:** writing review – edit article. **Charbel Badr:** methodology, writing review – edit article. **Lily Edwards:** writing review – edit article. **Reema Harrison:** conceptualisation, analysis, methodology, funding acquisition, project administration, supervision, writing original draft and review – editing.

## Funding

This work was supported by the 2023 MRFF Multidisciplinary Models of Primary Care Stream 2–3 (Project ID: 2035694).

## Disclosure

Not commissioned; externally peer reviewed.

*Artificial Intelligence (AI) Statement*: There was no use of AI tools in the writing of this manuscript.

## Conflicts of Interest

The authors declare no conflicts of interest.

## Supporting information


**Data S1:** Supplementary table and interviews.

## Data Availability

The de‐identified data we analysed are not publicly available, but requests to the corresponding author for the data will be considered on a case‐by‐case basis.

## References

[1] Australian Bureau of Statistics , “Patient Experiences, 2022–23; Table 5.3,” 2023, https://www.abs.gov.au/statistics/health/health‐services/patient‐experiences/2022‐23#data‐downloads.

[2] Australian Government. Department of Health, Disability and Ageing , “Strengthening Medicare Taskforce Report,” 2022, https://www.health.gov.au/resources/publications/strengthening‐medicare‐taskforce‐report?language=en.

[3] H. Sandvik , O. Hetlevik , J. Blinkenberg , and S. Hunskaar , “Continuity in General Practice as Predictor of Mortality, Acute Hospitalisation, and Use of Out‐Of‐Hours Care: A Registry‐Based Observational Study in Norway,” British Journal of General Practice 72 (2022): e84–e90. 10.3399/BJGP.2021.0340 PMC8510690 34607797

[4] Australian Government. Department of Health, Disability and Ageing , “About MyMedicare,” https://www.health.gov.au/our‐work/mymedicare/about.

[5] Australian Government. Department of Health, Disability and Ageing , “General Practice in Aged Care Incentive,” https://www.health.gov.au/our‐work/gpaci?language=en.

[6] S. M. Bates , J. Lin , L. Allen , M. Wright , and M. Kidd , “The Impact of Patient Enrolment in Primary Care on Continuity and Quality of Care Around the World 2014‐2024, and Lessons for Australia: A Scoping Review,” Medical Journal of Australia 222, no. 9 (2025): 462–471, https://www.mja.com.au/journal/2025/222/9/impact‐patient‐enrolment‐primary‐care‐continuity‐and‐quality‐care‐around‐world. 10.5694/mja2.52648 PMC12088321 40231620

[7] S. M. Bates , M. Wright , P. Brown , et al., “Characteristics of Patient Enrolment Policies in Primary Care: A Qualitative Analysis of 15 Schemes From 12 High‐Income Countries,” Lancet Primary Care 1, no. 2 (2025): 100023.

[8] B. Whitehead , “What Voluntary Patient Enrolment Means for GPs and Their Patients,” MJA InSight, 2023, https://insightplus.mja.com.au/2023/47/what‐voluntary‐patient‐enrolment‐means‐for‐gps‐and‐their‐patients.

[9] “AMA Position Statement on the Medical Home 2021,” https://www.ama.com.au/articles/medical‐home‐2021.

[10] J. Pearse , D. Mazevska , P. McElduff , et al., “Health Care Homes Trial Final Evaluation Report, Volume 1: Summary Report,” Health Policy Analysis. Australian Government Department of Health, 2022, https://www.health.gov.au/resources/publications/evaluation‐of‐the‐health‐care‐homes‐trial‐final‐evaluation‐report‐2022?language=en.

[11] D. T. Tran , M. O. Falster , J. Pearse , et al., “The Australian Health Care Homes Trial: Quality of Care and Patient Outcomes. A Propensity Score‐Matched Cohort Study,” Medical Journal of Australia 220, no. 7 (2024): 372–378, https://www.mja.com.au/journal/2024/220/7/australian‐health‐care‐homes‐trial‐quality‐care‐and‐patient‐outcomes‐propensity. 10.5694/mja2.52266 38514449

[12] R. Harrison , A. Chauhan , R. Mitchell , et al., “Evaluating Australia's “MyMedicare” Voluntary Patient Registration System: The Prospective Evaluation of Patient Registration Study Protocol,” Frontiers in Health Services 6 (2026): 1746024, 10.3389/frhs.2026.1746024. PMC13044130 41939296

[13] D. R. Thomas , “A General Inductive Approach for Analyzing Qualitative Evaluation Data,” American Journal of Evaluation 27, no. 2 (2006): 237–246.

[14] Australian Government. Department of Health, Disability and Ageing , “Information for MyMedicare General Practices and Healthcare Providers,” https://www.health.gov.au/our‐work/mymedicare/practices‐and‐providers#benefits.

[15] M. Sekhorn , M. Cartwright , and J. J. Francis , “Acceptability of Healthcare Interventions: An Overview of Reviews and Development of a Theoretical Framework,” BMC Health Services Research 17, no. 1 (2017): 88. 10.1186/s12913-017-2031-8 PMC5267473 28126032

[16] L. J. Damschroder , C. M. Reardon , M. A. Opra Widerquist , and J. Lowery , “Conceptualizing Outcomes for Use With the Consolidated Framework for Implementation Research (CFIR): The CFIR Outcomes Addendum,” Implementation Science 17, no. 1 (2022): 7. 10.1186/s13012-021-01181-5 PMC8783408 35065675

[17] A. G. Pereira and S. D. Pearson , “Patient Attitudes Toward Continuity of Care,” Archives of Internal Medicine 163, no. 8 (2003): 909–912. 10.1001/archinte.163.8.909 12719199

[18] H. Schers , S. Webster , H. van den Hoogen , et al., “Continuity of Care in General Practice: A Survey of Patients' Views,” British Journal of General Practice 52, no. 479 (2002): 459–462. PMC1314320 12051209

[19] T. Stokes , C. Tarrant , A. G. Mainous , et al., “Continuity of Care: Is the Personal Doctor Still Important? A Survey of General Practitioners and Family Physicians in England and Wales, the United States, and The Netherlands,” Annals of Family Medicine 3, no. 4 (2005): 353–359. 10.1370/afm.351 PMC1466891 16046569

[20] H. Thomas , M. Best , and G. Mitchell , “Health Care Homes and Whole‐Person Care: A Qualitative Study of General Practitioners' Views,” Australian Journal of General Practice 48, no. 12 (2019): 867–874. 10.31128/AJGP-05-19-4932 31774992

[21] C. L. Jackson and S. J. Hambleton , “Australia's Health Care Homes: Laying the Right Foundations,” Medical Journal of Australia 206, no. 9 (2017): 380–381, https://www.mja.com.au/journal/2017/206/9/australias‐health‐care‐homes‐laying‐right‐foundations. 10.5694/mja16.01470 28490291

[22] M. Wright , J. Hall , K. Van Gool , and M. Haas , “How Common Is Multiple General Practice Attendance in Australia?,” Australian Journal of General Practice 47, no. 5 (2018): 289–296. 10.31128/AJGP-11-17-4413 29779298

[23] J. Lin , “MyMedicare Promises Better Health Care. But Only 1 in 10 Patients Has Signed Up,” The Conversation, 2025, https://theconversation.com/mymedicare‐promises‐better‐health‐care‐but‐only‐1‐in‐10‐patients‐has‐signed‐up‐253335.

[24] MBS Online Australian Government , “Medicare Benefits Schedule – Note AN.0.47,” https://www9.health.gov.au/mbs/fullDisplay.cfm?type=note&q=AN.0.47&qt=noteID&criteria=965.

[25] Department of Health, Disability and Ageing , “Budget 2023–24: Building a Stronger Medicare [Media Release],” 2023, https://www.health.gov.au/ministers/the‐hon‐mark‐butler‐mp/media/budget‐2023‐24‐building‐a‐stronger‐medicare?language=en.

[26] Eastern Melbourne Primary Health Network , “Current MyMedicare Statistics,” 2024, https://emphn.org.au/news/current‐mymedicare‐statistics/.

[27] M. Wright and R. Versteeg , “Introducing General Practice Enrolment in Australia: The Devil Is in the Detail,” Medical Journal of Australia 216, no. 3 (2022): 159, https://www.mja.com.au/journal/2021/214/9/introducing‐general‐practice‐enrolment‐australia‐devil‐detail. 10.5694/mja2.51393 35112715

[28] A. Scott , T. Taylor , G. Russell , and M. Sutton , “Associations Between Corporate Ownership of Primary Care Providers and Doctor Wellbeing, Workload, Access, Organizational Efficiency, and Service Quality,” Health Policy 142 (2024): 105028, 10.1016/j.healthpol.2024.105028. 38387240

[29] Royal Australian College of General Practitioners , General Practice: Health of the Nation 2025 (RACGP, 2025), https://www.racgp.org.au/general‐practice‐health‐of‐the‐nation.

