# Supplementary material for: General Practice Staff and Consumer Views of Voluntary Patient Registration in Australia Under the MyMedicare Scheme: A Qualitative Study
Source: Med J Aust. 2026 Oct 30;224(11):e70318. doi: 10.5694/mja2.70318 (PMC13720891; doi:10.5694/mja2.70318)
Supplement: Supplementary file 1 [file MJA2-224-0-s001.pdf]

Supporting Information

Supplementary material

**This appendix was part of the submitted manuscript and has been peer reviewed.
It is posted as supplied by the authors.**

Appendix to: S. Vagholkar, A. Chauhan, G. Arnolda, et al. General Practice Staff and Consumer Views of Voluntary Patient Registration in Australia Under the MyMedicare Scheme: A Qualitative Study. *Medical Journal of Australia* 2026; doi: 10.5694/mja2.70318.

TABLE S1. Consolidated criteria for reporting qualitative studies (COREQ): 32-item checklist.

Developed from:
Tong A, Sainsbury P, Craig J. Consolidated criteria for reporting qualitative research (COREQ): a 32-item checklist for interviews and focus groups. International Journal for Quality in Health Care. 2007. Volume 19, Number 6: pp. 349 – 357

Item No	Guide Questions/Description	Reported on Page #/Comment
Domain 1: Research team and reflexivity		
Personal Characteristics		
1. Interviewer/ facilitator	Which author/s conducted the interview or focus group?	Pg 5
2. Credentials	What were the researcher's credentials? E.g., PhD, MD	Pg 5
3. Occupation	What was their occupation at the time of the study?	Pg 5
4. Gender	Was the researcher male or female?	Pg 5
5. Experience and training	What experience or training did the researcher have?	Pg 5
Relationship with participants		
6. Relationship established	Was a relationship established prior to study commencement?	No
7. Participant knowledge of the interviewer	What did the participants know about the researcher? e.g. personal goals, reasons for doing the research?	Information from PICF
8. Interviewer characteristics	What characteristics were reported about the interviewer/facilitator? e.g. Bias, assumptions, reasons and interests in the research topic	Pg 5
Domain 2: study design		
Theoretical framework		
9. Methodological orientation and Theory	What methodological orientation was stated to underpin the study? e.g. grounded theory, discourse analysis, ethnography, phenomenology, content analysis	Pg 4
Participant selection		
10. Sampling	How were participants selected? e.g., purposive, convenience, consecutive, snowball	Pg 4
11. Method of approach	How were participants approached? e.g., face-to-face, telephone, mail, email	Pg 4
12. Sample size	How many participants were in the study?	Pg 6
13. Non-participation Setting	How many people refused to participate or dropped out? Reasons?	Unable to comment as voluntary option to be interviewed
14. Setting of data collection	Where was the data collected? e.g., home, clinic, workplace	Pg 5

Item No	Guide Questions/Description	Reported on Page #/Comment
15. Presence of nonparticipants	Was anyone else present besides the participants and researchers?	No
16. Description of sample	What are the important characteristics of the sample? e.g. demographic data, date	Pg 6,15,16
Data collection		
17. Interview guide	Were questions, prompts, and guides provided by the authors? Was it pilot tested?	Pg 5 Supporting information
18. Repeat interviews	Were repeat interviews carried out? If yes, how many?	No
19. Audio/visual recording	Did the research use audio or visual recording to collect the data?	Pg 5
20. Field notes	Were field notes made during and/or after the interview or focus group?	Yes
21. Duration	What was the duration of the interviews or focus group?	Pg 5
22. Data saturation	Was data saturation discussed?	Pg 11
23. Transcripts returned	Were transcripts returned to participants for comment and/or correction?	No
Domain 3: analysis and findings		
Data analysis		
24. Number of data coders	How many data coders coded the data?	Pg 5
25. Description of the coding tree	Did the authors provide a description of the coding tree?	No
26. Derivation of themes	Were themes identified in advance or derived from the data?	Pg 5
27. Software	What software, if applicable, was used to manage the data?	Pg 5
28. Participant checking	Did participants provide feedback on the findings?	No
Reporting		
29. Quotations presented	Were participant quotations presented to illustrate the themes/findings? Was each quotation identified? e.g., participant number	Pgs 6-9 Pgs 17-20 Table 3
30. Data and findings consistent	Was there consistency between the data presented and the findings?	Pgs 6-9 Pgs 17-20
31. Clarity of major themes	Were major themes clearly presented in the findings?	Pgs 6-9
32. Clarity of minor themes	Is there a description of diverse cases or a discussion of minor themes?	Pgs 6-9

SECTION S1. Topic Guide: Consumer interviews.

Interviewer: Introduce evaluation project, briefly explain MyMedicare, confirm consent to audio record and participate, check for any questions and respond.

1. <u>CFIR Innovation, outer setting domains – MyMedicare</u>

   1.1. Before you were invited to take part in this project, what did you know about MyMedicare?
   1.2. From what you know about MyMedicare now, what are your initial impressions of this initiative?
   1.3. How well do you think the MyMedicare initiative meets the needs of people accessing care in the Australian health system?
      a) What might work well for you?
      b) What might benefit you from having MyMedicare?
      c) What might not work so well for you?
      d) What are you unsure about / need to know more about?

2. <u>CFIR Inner setting and individuals domains</u>

Interviewer: Outline the intended benefits of MyMedicare for continuity of care e.g. having a single healthcare provider should mean they know more about your overall health and can provide more suitable advice and care, access to longer telehealth for certain conditions.

   2.1. When you think about going to see your doctor, what would need to happen for you to experience these types of benefits from MyMedicare?
   2.2. What do you think might make it difficult for you to experience these types of benefits?

3. <u>CFIR Implementation process domain – MyMedicare + (Theoretical Framework of Acceptability (TFA) & Proctor et al.'s implementation outcomes)</u>

   3.1. Are you registered with MyMedicare? (adoption)

   If yes:

      a) Where did you find out about MyMedicare?
      b) What kind of information have you seen?
      c) Why did you decide to register? (affective attitude/ adoption)
      d) How easy was it to register? (burden)
      e) What support did you need to register and was this available? (burden)
      f) What additional work or thinking was required from you or your health professional to register for MyMedicare? (burden/costs/ intervention coherence)

   If no:

      g) How do you feel about registering for the MyMedicare scheme? (affective attitude/acceptability)
      h) Why did you decide not to register so far? (affective attitude/adoption)

i) What additional work or thinking would be required from you or your health professional to register for and use MyMedicare? (burden)
j) If not heard, what would have helped you to know that MyMedicare was being introduced?

3.2. How successful do you think the information about MyMedicare has been in helping people to know about the initiative and use it?
   a) What else needs to happen?

3.3. To your knowledge, have you or your health professionals used MyMedicare for your care?
   a) How has using MyMedicare changed your care? (effectiveness)
   b) How has using MyMedicare changed your relationship with the main person that provides your care e.g. your GP or primary care professional? (effectiveness)

4. Is there anything else you would like to add about MyMedicare in the Australian health system?

SECTION S2. Topic Guide: Policy and service delivery stakeholder interviews.

Interviewer: Introduce evaluation project, briefly explain MyMedicare, confirm consent to audio record and participate, check for any questions and respond, tell me about about yourself – establish consumer, health professional, system stakeholder role.

5. <u>CFIR Innovation domain - MyMedicare</u>

What are your initial impressions of the concept of MyMedicare?

- To what extent do you think evidence from other contexts support this type of model?
- Where does MyMedicare fit in relation to primary care reform and how effective do you anticipate this approach to be?
- How well does MyMedicare fit with the current system for accessing primary care / streamline existing approaches?
- Do you think MyMedicare has the potential to improve patient care? If not, why not? What could be done to make it a more useful/impactful program?

6. <u>CFIR Outer setting domain</u>

To what extent does MyMedicare provide a suitable approach for the Australian health context?

- In what ways does our health system support or challenge its implementation?
- How well can MyMedicare work in a federated (primary care funded by Commonwealth and hospitals funded by state governments who receive Commonwealth funding via the National Health Reform Agreement) model?
- How well can MyMedicare work within current legislation, professional guidelines and funding models?

7. <u>CFIR Inner setting and individuals domains</u>

To what extent does the health system infrastructure support the implementation of MyMedicare, for example is the necessary patient health data available to providers?

- What are the technological enablers and what is needed? (provide examples or clarifying intention statement as required)
- How will different parts of the health system have to connect to make this work?
- What cultural change might be needed in the health system to enable MyMedicare to be impactful? (provide examples or clarifying intention statement as required)
- How does MyMedicare fit with current workflows, systems and processes?
- What resources will be needed and how well are we currently equipped as a system?
- Who must be engaged for MyMedicare implementation, delivery, and/or use?
- What information or training and skill development will staff and consumers require for MyMedicare to be used?
- How well do you understand MyMedicare and what it is aiming to provide for practices and patients? If limited understanding, why do you think that is?

8. CFIR Implementation process domain

How has MyMedicare been implemented so far?

- What steps have been taken to encourage the adoption and use of MyMedicare?
- How successful has the implementation been so far?
- To what extent have implementation activities been tailored to meet stakeholder needs?
- What else needs to happen?
- Is there anything else you would like to add about the implementation of MyMedicare in the Australian health system?

For GPs and practice staff (this might fit into the additional topics below or be in this implementation section)

- How has your practice approached implementing MyMedicare?
- How have you found communicating to patients about MyMedicare? What mechanisms has the practice used – GP talking to patient, practice nurse talking to patient, written information?
- What is the response of patients when MyMedicare is discussed with them?
- Is the practice registering patients or asking patient to register themselves online or a combination? How have you found this process? Challenges?
- What impact has patient registration had on practice workload?

Additional topics for those delivering MyMedicare in practice (Theoretical Framework of Acceptability (TFA) & Proctor et al.'s implementation outcomes)

9. How do you feel about registering patients for the MyMedicare scheme? (affective attitude)
10. What additional work or thinking is required from you and the patient in order to register for and use MyMedicare? – consider differences by context and cohort. (burden/feasibility)
11. How consistently are you able to invite, discuss and check that patients have enrolled in MyMedicare? (fidelity)
12. At this stage, how are you using MyMedicare to improve patient care? (perceived effectiveness)
13. At this stage, how effective has MyMedicare been in improving continuity of care? (perceived effectiveness)
14. To what extent has the experience of registering and using MyMedicare been equitable for different care contexts and population groups? (ethicality /intervention coherence)
15. What time and resources are you using to register patients and use MyMedicare? (costs & opportunity costs) – consider here using patient data for quality improvement in their care.
16. In taking the time to register patients for MyMedicare, what are the opportunity costs for care providers? (opportunity costs)
17. How confident do you feel registering and using MyMedicare with your patients? (self-efficacy)
